# β-Elemene Triggers ROS-dependent Apoptosis in Glioblastoma Cells Through Suppressing STAT3 Signaling Pathway

**DOI:** 10.3389/pore.2021.594299

**Published:** 2021-03-25

**Authors:** Shi-zhong Cai, Qian-wei Xiong, Li-na Zhao, Yi-ting Ji, Zheng-xiang Luo, Zhou-rui Ma

**Affiliations:** ^1^ Department of Child and Adolescent Healthcare, Children’s Hospital of Soochow University, Suzhou, China; ^2^ Department of Surgery, Children’s Hospital of Soochow University, Suzhou, China; ^3^ Department of Laboratory Medicine, Key Laboratory of Clinical Immunology of Jiangsu Province, The First Affiliated Hospital of Soochow University, Suzhou, China; ^4^ Department of Neurosurgery, Nanjing Brain Hospital Affiliated to Nanjing Medical University, Nanjing, China

**Keywords:** β-elemene, glioblastoma, apoptosis, stat3, ROS

## Abstract

Glioblastoma is one of the most aggressive primary brain tumors with few treatment strategies. β-Elemene is a sesquiterpene known to have broad spectrum antitumor activity against various cancers. However, the signaling pathways involved in β-elemene induced apoptosis of glioblastoma cells remains poorly understood. In this study, we reported that β-elemene exhibited antiproliferative activity on U87 and SHG-44 cells, and induced cell death through induction of apoptosis. Incubation of these cells with β-elemene led to the activation of caspase-3 and generation of reactive oxygen species (ROS). Western blot assay showed that β-elemene suppressed phosphorylation of STAT3, and subsequently down-regulated the activation of *p*-JAK2 and *p*-Src. Moreover, pre-incubation of cells with ROS inhibitor *N*-acetyl-_L_-cysteine (NAC) significantly reversed β-elemene-mediated apoptosis effect and down-regulation of JAK2/Src-STAT3 signaling pathway. Overall, our findings implied that generation of ROS and suppression of STAT3 signaling pathway is critical for the apoptotic activity of β-elemene in glioblastoma cells.

## Introduction

Glioblastoma (GBM) is one of the most aggressive malignancies with high recurrence rate and low survival rate [1]. Temozolomide (TMZ) is the first-line chemotherapeutic strategy for the treatment of GBM currently. Although TMZ has been proven to effectively inhibit GBM proliferation, intrinsic and acquired resistance in GBM cells ultimately limits its efficacy [2]. Development of novel chemotherapeutic drugs for GBM is hampered by many difficulties, like drug resistance, toxicity, disability to cross the blood-brain barrier [3]. Thus, research and development of new drugs with promising therapeutic effects and lower toxicity for GBM treatment is urgently needed [4]. Great efforts have been made to understand the basic biology of GBM and many molecular signaling pathways have been identified as potential therapeutic targets for this devastating cancer [5].

Up-regulation and hyperactivation of signal transducer and activator of transcription 3 (STAT3) in GBM have been identified by numerous studies, and aberrant STAT3 activation is associated with poor prognosis [6]. Seven different STATs (1, 2, 3, 4, 5a, 5b, and 6) have been found in mammalian cells until now, and of all the STATs, STAT3 is certainly the most eminent among cancers. It can be activated by a variety of stimuli, including cytokines, growth factors and interferons [7]. In a high percentage of GBM cells, persistent activation of STAT3 induces cell proliferation, anti-apoptosis, glioma stem cell maintenance, tumor invasion and immune evasion [8]. Thus, STAT3 is a potential therapeutic target of GBM, and many small molecular inhibitors targeting STAT3 have been developed as effective strategies for the treatment of GBM [9].

β-Elemene, a sesquiterpene, is the main component of elemene, which was extracted from the traditional Chinese medicinal herb *Curcuma wenyujin* [10]. β-Elemene is renowned for its anticancer activity against various of cancers by inhibiting cell proliferation, arresting cell cycle, inducing an apoptotic trigger, downregulating anti-apoptotic signals, decreasing mitochondrial potential, and enhancing the activity of the immune system [11]. For example, β-elemene has been widely studied for the treatment of breast cancer [12], lung cancer [13], brain tumor [14], and other malignant tumors. In addition, a number of novel β-elemene derivatives exhibiting potent anti-glioma activity have been reported by structural modification of β-elemene [15]. These studies suggest that β-elemene might be a precious candidate for future anticancer medications to treat GBM.

In this study, we found that β-elemene inhibited the proliferation of U87 and SHG-44 cells. β-Elemene triggers ROS-dependent apoptosis in glioblastoma cells through suppressing STAT3 signaling pathway, it suppressed phosphorylation of STAT3, and subsequently down-regulated the activation of *p*-JAK2 and *p*-Src.

## Materials and Methods

### Cell Lines and Cell Culture

The glioma cell lines SHG-44 and U87 were purchased from the Type Culture Collection of the Chinese Academy of Sciences (Shanghai, China). All cells were cultured in Dulbecco’s modified Eagle’s medium (DMEM) supplemented with 10% fetal bovine serum (FBS), 50 IU/ml penicillin and 50 mg/ml streptomycin (Gibco, Grand Island, NY, United States) under the conditions of 37°C in a humidified atmosphere with 5% CO_2_.

### Reagents

β-Elemene was purchased from Dalian Jingan Pharmaceutical Co. China (Purity 99.5%). MTT (3-(4,5)-dimethylthiahiazo (-z-y1)-3,5-di-phenytetrazoliumromide), DCF-DA (2,7-dichlorodihydrofluorescein diacetate), NAC (N-Acetyl-L-cysteine) were purchased from Sigma-Aldrich (St. Louis, MO, United States).

### Cell Viability Assay

Cell viability was detected using MTT assay. 5 × 10^3^ cells were seeded into 96-well plates, after incubation for 24 h, the cells were treated with the indicated concentrations of β-elemene for 24, 48, or 72 h. Then, 10 *μ*l of 0.5 mg/ml MTT was added and the mixture was incubated at 37°C for another 4 h. The culture medium was removed and 100 *μ*l DMSO was added to dissolve the formazan crystals. The absorbance at 490 nm was measured using a microplate reader (Bio-Tek, United States).

### Caspase-3 Activity Measurement

The activity of caspase-3 was detected using the Caspase-3 Activity Assay Kit (Beyotime, Jiangsu, China) according to the manufacturer’s recommendation. The absorbance at 405 nm was measured using a microplate reader after treatment with β-elemene for 12 h (Bio-Tek, Vermont, United States).

### Detection of Intracellular ROS

ROS levels were detected using probe 2,7-dichlorodihydrofluorescein diacetate (DCF-DA). In brief, cells were treated with different concentrations of β-elemene for 12 h with or without pre-incubation of 5 mM NAC for 30 min, then incubated with 10 μM DCF-DA for 20 min at 37°C. PBS was used to wash the cells for three times before detection. Fluorescence microscope (Olympus, Tokyo, Japan) was used for direct observation. To detect fluorescence intensity, cells were collected and fluorescence was detected with a flow cytometry (FACScan, BD Biosciences, United States).

### Annexin V-FITC/PI Assay

Annexin V-FITC/PI apoptosis detection kit was used to perform the apoptotic assay following the manufacturer’s instructions (Beyotime, Jiangsu, China). Briefly, after treatment, 1 × 10^6^ cells were washed using 1 ml binding buffer for three times, then centrifuged at 300 × g and stained with 10 μl Annexin V-FITC solution at 37°C for 15 min. Before detecting, 5 μl PI solution was added to the samples and the apoptotic cells were detected using flow cytometry (BD Biosciences, United States).

### Western Blot Analysis

Cells after treatment were lysed and centrifuged at 12,000 × *g* for 15 min. The supernatant was collected and the protein concentrations were detected using the BCA assay kit (Beyotime, Jiangsu, China). Equal amounts of total proteins (20–60 μg) were separated using SDS-polyacrylamide gel and electrotransferred to PVDF membranes. The membranes were initially blocked with 5% nonfat dry milk in PBS-Tween-20 (0.1%, v/v) at 4°C for 12 h and then probed with primary antibodies against cleaved caspase-3 (mouse IgG1), Bcl-2 (mouse IgG1 κ), Bax (mouse IgG1 κ), STAT3 (mouse IgG1 κ), p-STAT3 (mouse IgG1), *p*-JAK2 (Rabbit mAb), JAK2 (mouse IgG2b κ), *p*-Src (mouse IgG2a κ), Src (mouse IgG2a κ) or GAPDH (Rabbit mAb) (Santa Cruz, CA, United States), which were diluted following the manufacturer’s instructions at 4°C overnight. Then the membranes were blotted with the horseradish peroxidase-conjugated anti-mouse or anti-rabbit secondary antibodies (Beyotime, Jiangsu, China, 1:5,000) for 2 h at 37°C. Finally, the bands were visualized through the enhanced chemiluminescence protocol. Signals were densitometrically quantified and normalized to GAPDH. The results were analyzed using Image J software (NIH, United States).

### Statistical Analysis

All data were expressed as means ± SD of three replicates. Statistical comparisons were performed by Student’s *t*-test to compare two groups. To compare more than two groups, one-way ANOVA with Tukey *post hoc* analysis was performed. Statistical significance was defined as *p* < 0.05 between different groups, the analysis were performed using GraphPad Prism 5.0 software.

## Results

### β-Elemene Inhibited the Growth and Induced Apoptosis of Glioma Cells

**FIGURE 1:**
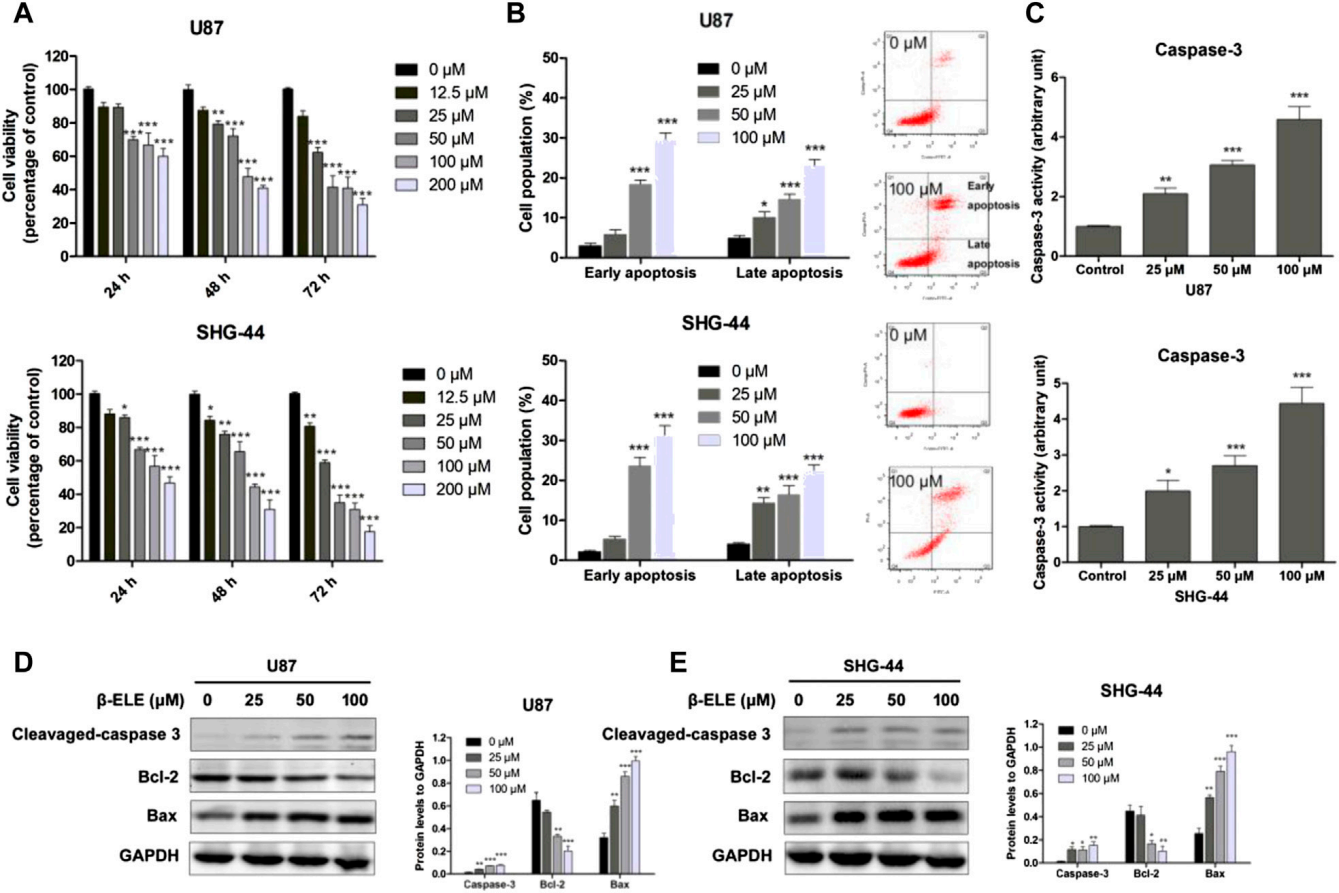
β-Elemene decreased cell viability of glioma cells and induced cell apoptosis. **(A)** U87 and SHG-44 cells were treated with various concentrations (0–200 μM) of β-elemene and incubated for indicated time (24, 48 and 72 h). Cell viability was determined with MTT method. **(B)** Cell apoptosis of glioma cells was determined by Annexin V/PI co-staining flow cytometric analysis after incubation with 0, 25, 50 and 100 μM β-elemene for 24 h. **(C)** Caspase-3 activity was determined after incubation with 0, 25, 50 and 100 μM β-elemene for 12 h **(D,E)** Expression levels of cleaved-caspase 3, Bcl-2, and Bax in U87 and SHG-44 cells after treatment with 0, 25, 50 and 100 μM β-elemene for 24 h. Data are presented as mean ± SD of three independent experiments, *p < 0.05, **p < 0.01, ***p < 0.001, compared with control group.

To verify the anticancer activity of β-elemene, the cell viabilities of U87 and SHG-44 cells were determined with MTT assay after incubation with various concentrations of β-elemene for 24, 48, and 72 h. It was found that β-elemene induced a dose- and time-dependent inhibition of the growth of glioma cells (Figure 1A). To determine whether β-elemene induced apoptosis of these cells, Annexin V/PI co-staining was performed and the cells at early and late stages were analyzed with flow cytomery. The results showed that β-elemene induced the apoptosis of glioma cells in a dose-dependent manner (Figure 1B). In addition, increased activity of caspase-3 in both U87 and SHG-44 cells after treatment with β-elemene was also observed (Figure 1C). The expression levels of cleaved caspase-3, Bcl-2, and Bax were also examined using western blot analysis in both U87 and SHG-44 cells, and it was found that β-elemene increased the expression of cleaved caspase-3 and Bax, while decreased the expression of Bcl-2 (Figures 1D,E).

### β-Elemene Induced ROS Generation in Glioma Cells

**FIGURE 2:**
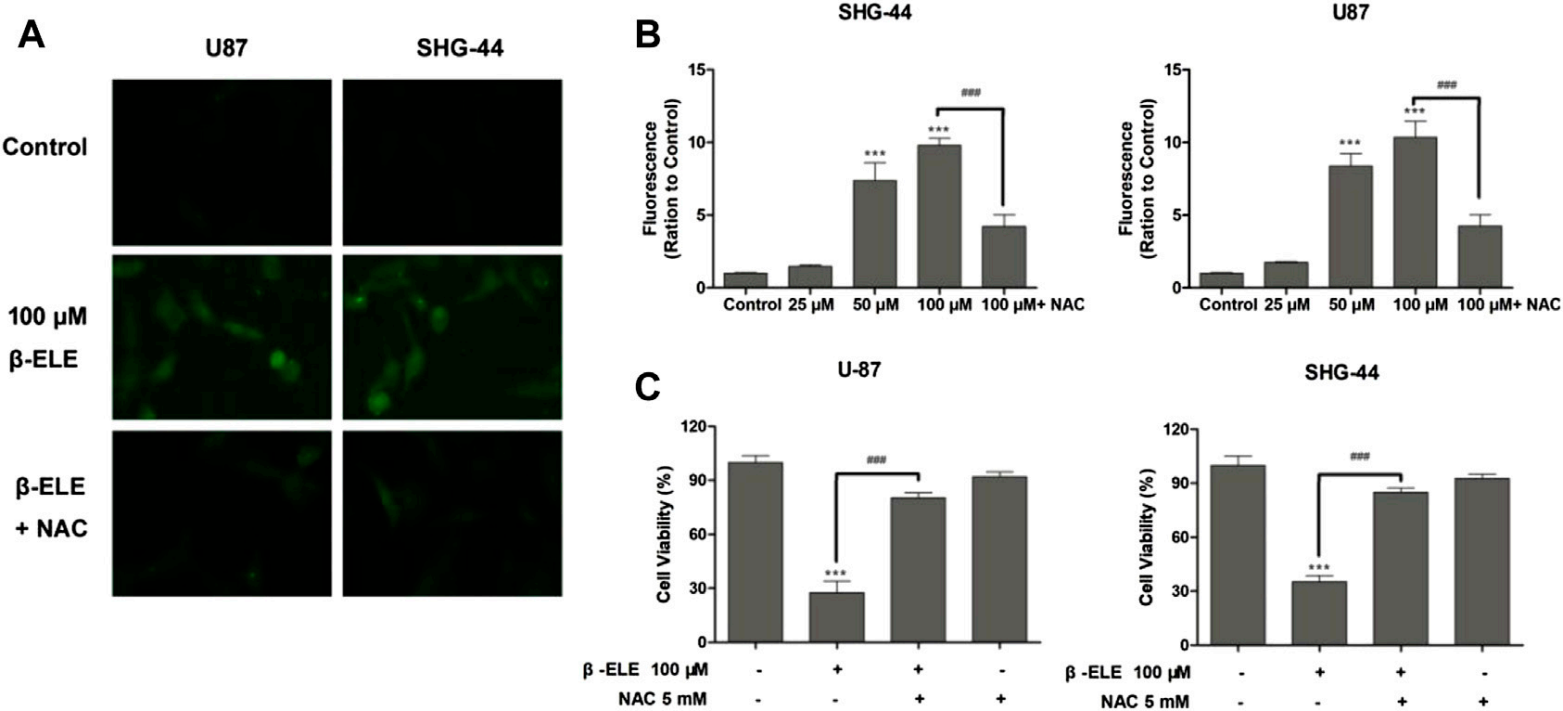
β-Elemene increased ROS production in glioma cells. **(A)** The levels of ROS in U87 and SHG-44 cells were indicated by DCF-DA staining, and green fluorescence intensity indicated the degree of ROS. **(B)** The levels of ROS in glioma cells after treatment with 0, 25, 50 and 100 μM β-elemene for 12 h were measured by flow cytometric analysis, pre-incubation of cells with 5 mM NAC for 0.5 h reduced the production of ROS. **(C)** The clearance of ROS partly increased cell viability. Cell viability was analyzed by the MTT assay when U87 or SHG-44 cells were treated with 100 μM β-elemene for 48 h or pre-incubated with 5 mM NAC for 0.5 h. Data are presented as mean ± SD of three independent experiments, ***p < 0.001, compared with control group, ^###^ *p* < 0.001, compared with NAC treated group.

DCF-DA, a common oxidative stress indicator, was used to measure ROS in glioma cells [16]. As shown in Figure 2A, after incubation with 100 μM β-elemene for 12 h, green fluorescence was observed under fluorescence microscopy in both U87 and SHG-44 cells, indicating the generation of ROS. However, pre-incubation with antioxidant NAC significantly attenuated the generation of ROS in these cells. When ROS production was detected with different doses of β-elemene by analysis of fluorescence intensity, it was found that β-elemene increased the generation of ROS in a dose-dependent manner in glioma cells Figure 2B. In addition, pre-incubation of NAC not only abolished the generation of ROS, but also the cytotoxicity of β-elemene (Figure 2C), confirming the critical role of ROS in β-elemene induced apoptosis.

### β-Elemene Suppressed Phosphorylation of STAT3 in Glioma Cells

**FIGURE 3:**
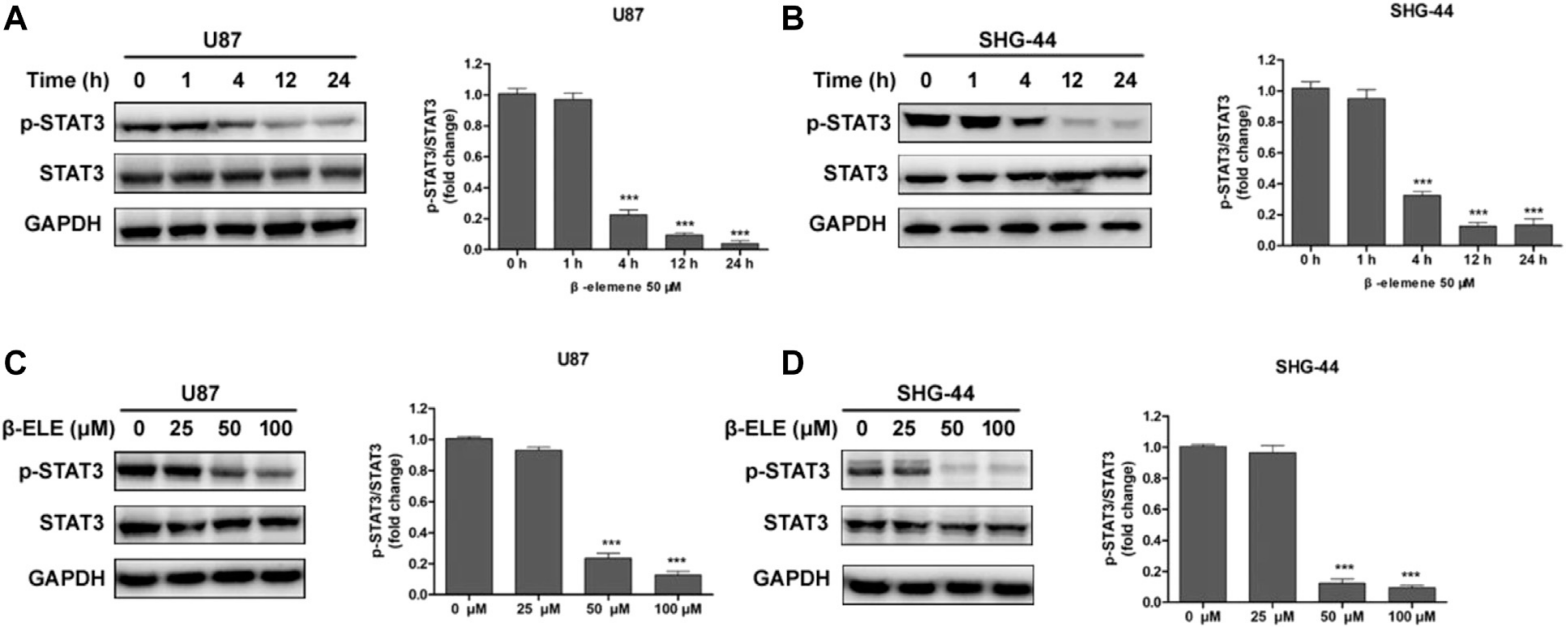
β-Elemene suppressed phosphorylation of STAT3 in glioma cells. **(A,B)** Western blotting showed that β-elemene (50 μM) reduced the phosphorylation of STAT3 in a time-dependent manner. The GAPDH was used as a control and the blots were quantitatively evaluated. **(C,D)** Western blotting showed that after treatment with β-elemene for 12 h reduced the phosphorylation of STAT3 in a dose-dependent manner. The bands corresponding to each protein were quantified using GAPDH as a control and normalized relative to band intensities in control group. Data are presented as mean ± SD of three independent experiments, ***p < 0.001, compared with control group.

To verify whether β-elemene affected the expression of STAT3, U87 and SHG-44 cells were incubated with 50 μM β-elemene for different time and the expression levels of STAT3 and phospho-STAT3 were detected by western blot. It was found that the expression level of p-STAT3 was decreased over time and STAT3 was not affected by β-elemene in either U87 or SHG-44 cells (Figures 3A,B). In addition, after incubation with 25, 50 and 100 μM β-elemene for 12 h, statistical difference of p-STAT3 expression was observed in 50 and 100 μM groups compared with control group (Figures 3C,D). These findings suggested that β-elemene suppressed the STAT3 activation in glioblastoma.

### β-Elemene Suppressed Phosphorylation of STAT3 via JAK2/Src Pathway

**FIGURE 4:**
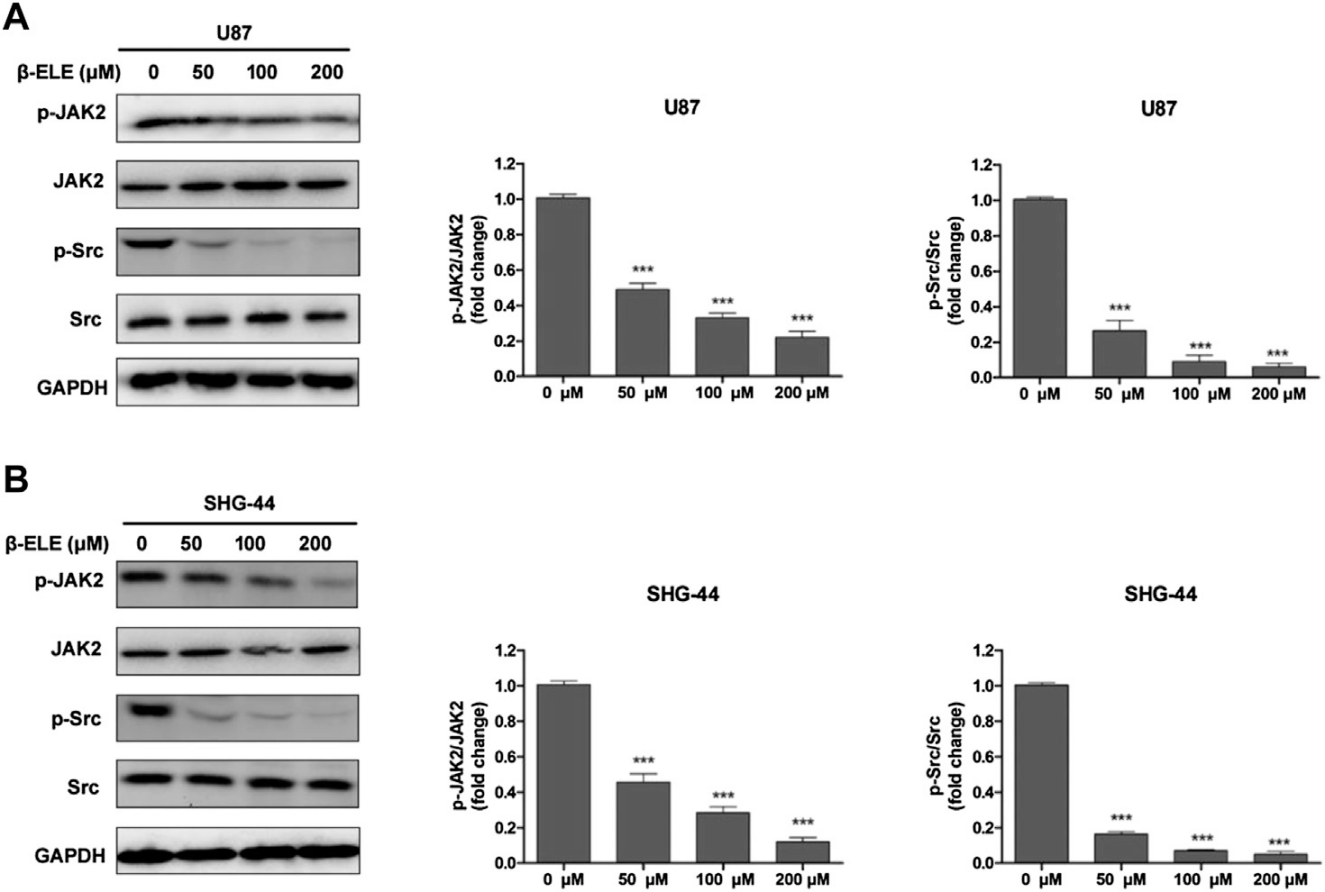
β-Elemene suppressed phosphorylation of JAK2 and Src in glioma cells. **(A)** Western blotting showed that β-elemene reduced the phosphorylation of JAK2 and Src in a dose-dependent manner in U87 cells and **(B)** SHG-44 cells. The bands corresponding to each protein were quantified using GAPDH as a control and normalized relative to band intensities in control group. Data are presented as mean ± SD of three independent experiments, ***p < 0.001, compared with control group.

It has been reported that STAT3 can be activated by Janus-activated kinases (JAKs) and c-Src [17], thus, we evaluated the expression levels of JAK2 and Src in glioma cells after incubation with β-elemene by western blot analysis. The results showed that β-elemene dose-dependently decreased the phosphorylation of JAK2 and Src in both U87 and SHG-44 cells, while the expression levels of JAK2 and Src were not affected (Figures 4A,B).

### STAT3 Activation Is Mediated by ROS Production in Glioma Cells

**FIGURE 5:**
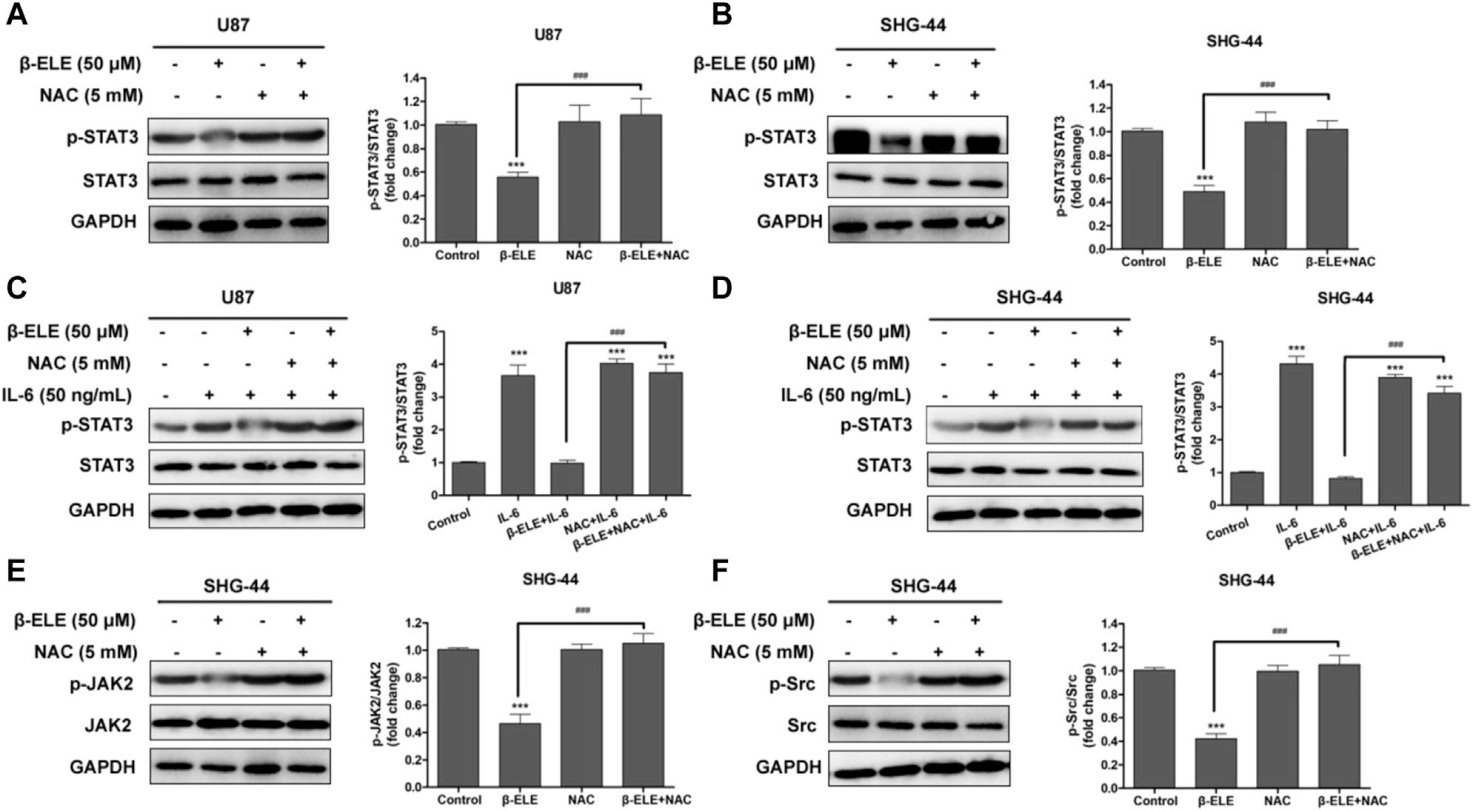
ROS has a great effect on β-elemene-induced suppression of STAT3 signaling in glioma cells. **(A,B)** Cells were treated with 50 μM β-elemene in the absence or presence of 5 mM NAC for 24 h, and cell lysates were subjected to western blot to analyze the expression of p-STAT3 and STAT3. **(C,D)** Cells were pre-treated with 5 mM NAC, and then IL-6 and β-elemene were added and incubated for 24 h. Cell lysates were subjected to western blot to analyze the expression of p-STAT3 and STAT3. **(E,F)** SHG-44 cells were treated with 50 μM β-elemene in the absence or presence of 5 mM NAC for 24 h, and cell lysates were subjected to western blot to analyze the expression of p-JAK2, JAK2, p-Src, and Src. GAPDH was used as a control and the blots were quantitatively evaluated. Data are presented as mean ± SD of three independent experiments, ***p < 0.001, compared with control group, ^###^ *p* < 0.001, compared with NAC treated group.

In order to identify the association between ROS production and STAT3 activation, U87 and SHG-44 cells were pre-treated with NAC and the expression levels of STAT3 and p-STAT3 were detected. It was found that pre-incubation of NAC significantly abolished the β-elemene induced decrease of expression level of p-STAT in both U87 and SHG-44 cells, while the expression level of STAT3 was not affected (Figures 5A,B). It is reported that when cancer-related inflammatory cytokine interleukin (IL)-6 binds to its receptor, STAT3 would be activated [18]. Thus, we further tested whether the activation of STAT3 by IL-6 would be influenced by β-elemene. It was found that exposure of glioma cells to IL-6 (50 ng/ml) increased the expression of p-STAT3, while co-incubation of β-elemene abolished such activation. However, NAC pre-incubation dramatically attenuated the activity of β-elemene to suppress IL-6-induced STAT3 activation (Figures 5C,D). In addition, β-elemene induced decline of expression levels of *p*-JAK2 and p-Src was also reversed by NAC pre-incubation in both U87 and SHG-44 cells (Figures 5E,F). Overall, these results indicated that β-elemene suppressed the activation of STAT3 pathway through elevated ROS production in glioma cells.

## Discussion

Current therapeutic regimens for GBM are mainly based on surgery, chemotherapy and radiotherapy. Bioactive phytochemicals are attracting increasing attention for the prevention and treatment of GBM due to their low toxicity and unique mechanism of actions. Many natural products have been reported to exhibit potent antitumor activities against GBM, such as curcumin [19], β-elemene [20], quercetin [21], and phloretin [22]. β-Elemene has been widely reported to have potent antitumor activities in various cancer cell lines. Particularly, β-elemene exhibited potent *in vitro* and *in vivo* antitumor effects in glioma cells [23]. In China, β-elemene has been used in clinical practice as an adjuvant medicine in the treatment of GBM. However, the detailed antitumor mechanism of β-elemene in glioma cells remains unclear. Illumination of its mechanism would accelerate its further development as an antitumor agent for the treatment of GBM.

Apoptosis is a specific and programmed mechanism of cell death which regulates homeostasis of tissues and elimination of malignant cells. The apoptotic process is involved in the mechanism of many natural products, including β-elemene. In this study, we found that β-elemene decreased cell viability and induced cell apoptosis of U87 and SHG-44 glioma cells. β-Elemene induced apoptosis in glioma cells was associated with the activation and cleavage of caspase-3. Jiang and co-workers also reported that β-elemene induced apoptosis in human glioma cells by regulation of Fas/FasL and activation of caspase-3, -8 and -9 [24]. Shi and co-workers showed that β-elemene induced glioma cell apoptosis by downregulating survivin and its interaction with hepatitis B X-interacting protein [25].

Many factors would induce apoptosis in cancer cells, and numerous studies have illustrated that excessive accumulation of intracellular ROS is a signal for initiating apoptosis [26]. Many natural products have been reported to induce apoptosis of cancer cells through generation of ROS. For example, ouabain is reported to elicit human glioblastoma cells apoptosis by generating ROS in ERK-p66SHC-dependent pathway [27]. Huang and co-workers reported that phloretin induces cell cycle arrest and apoptosis of human glioblastoma cells through the generation of ROS [22]. However, the exact role of ROS in cells is controversial due to its action as a double-edged sword. At low levels, ROS promotes tumor development through activating signaling pathways to accelerate the proliferation and differentiation of cells, while at high levels, ROS is toxic and results in oxidative damage and cell death. Cancer cells often have increased oxidative stress compared with normal cells and are more vulnerable to ROS insults. Accumulation of excessive ROS resulted in oxidative DNA damage, mitochondrial dysfunction, and enzyme inactivation, making it an important target for development of anticancer drugs. The present study showed that β-elemene induced ROS generation in U87 and SHG-44 cells in a dose- and time-dependent manner. In addition, β-elemene induced ROS generation could be blocked by an antioxidant, NAC. Similarly, the apoptotic activity of β-elemene is also ROS-dependent, pre-incubation of U87 and SHG-44 cells with NAC abolished its apoptotic activity. These results suggested that ROS generation is critical in β-elemene induced antitumor activity in GBM cells.

STAT3 is a cytosolic transcription factor which regulates angiogenesis, proliferation and survival of cells. Constitutive activation or aberrant phosphorylation of STAT3 are often identified in many human cancer cells, including breast lung, prostate and glioblastoma [28]. Phosphorylated STAT3 is dimerized and transferred into the nucleus to further regulate genes involved in cell survival, proliferation, immune regulation and invasion. Targeting STAT3 signaling by small molecules might be a potential strategy for the treatment of human cancer. In this study, we found that β-elemene inhibited the growth of glioblastoma cells and induced apoptosis, accompanied by remarkable suppression of active STAT3 in a dose- and time-dependent manner. In addition, we further explored the association between ROS generation and STAT3 activation. The results showed that β-elemene induced STAT3 activation was blocked by an antioxidant, NAC, which is a ROS inhibitor, suggesting that β-elemene abrogated STAT3 activation via ROS-mediated oxidative damage and then induced apoptosis in glioblastoma cells.

Overall, our data suggest that β-elemene can inhibit the growth of glioblastoma cells through suppressing STAT3 signaling pathway, which is mainly regulated by ROS-mediated oxidative stress. Our study implied that β-elemene could be developed as a potential antitumor agent for the treatment of GBM through targeting STAT3.

## Data Availability

The original contributions presented in the study are included in the article/Supplementary Material, further inquiries can be directed to the corresponding authors.
